# A Rare Case of Isolated Right Ventricular Non-compaction With the Novel *TTN* Mutation

**DOI:** 10.3389/fcvm.2022.845973

**Published:** 2022-04-29

**Authors:** Piao-piao Huang, Ya-xin Tang, Xian-sheng Huang

**Affiliations:** Department of Cardiovascular Medicine, The Second Xiangya Hospital, Central South University, Changsha, China

**Keywords:** non-compaction, ventricular tachycardia, *TTN*, spongy heart, trabeculae

## Abstract

Isolated right ventricular non-compaction (RVNC) is rare yet life-threatening if left untreated, especially when accompanied by ventricular tachycardia. We describe a rare case of isolated RVNC, presenting as a prominent and excessive trabeculation of the right ventricle (RV), with an abnormal electrocardiogram. The transthoracic echocardiography, computed tomography, and ventricular angiography results clearly demonstrated an isolated spongy RV, both anatomically and functionally. Genetic testing identified a missense mutation of *TTN*. Combined, the diagnosis of RVNC was established. The subsequent combination of heart failure therapy, antiarrhythmic, and anticoagulation therapy were effective with a favorable outcome. This case report describes the possible etiology, manifestation, characteristic images, and problematic diagnostic criteria of the isolated RVNC. This case also emphasizes the necessity for comprehensive cardiac screening in familial cardiomyopathy.

## Introduction

Ventricular non-compaction remains a genetically and phenotypically heterogeneous myocardial disorder with multiple possible concomitant phenotypes (1). Pathologically, it is characterized by excessive trabeculae and deep intertrabecular recesses in the ventricle. Non-compaction refers to the cessation of compaction of the loosely interwoven meshwork of myocardial fibers during intrauterine life, typically occurring in the left ventricle or bi-ventricle, and isolated RVNC is rare. The pathogenesis of ventricular non-compaction remains unclear. Genetics is believed to play an important role since genetic defects account for almost 40% of patients with ventricular non-compaction. However, studies have demonstrated that acquired causes are also expected, specifically in sporadic adults (2). Symptomatic individuals with ventricular non-compaction present varying degrees of heart failure, systemic thromboembolism, arrhythmia, or sudden cardiac death (SCD). Here, we present a case of an isolated RVNC, in which the first manifestation was syncope caused by VT.

## Case Description

**FIGURE 1:**
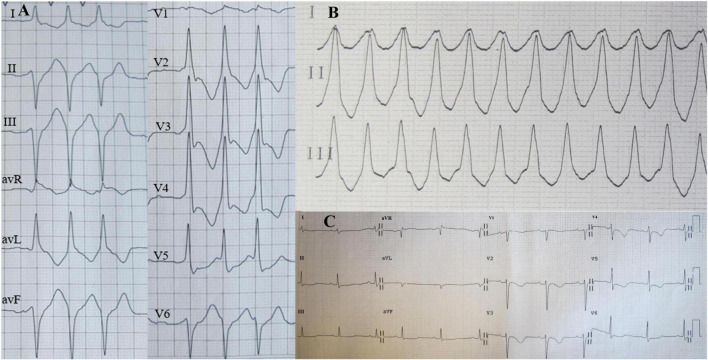
Holter electrocardiogram (ECG) on admission showed three consecutive episodes of ventricular premature beats **(A)** and ventricular tachycardia (VT) **(B)**; Twelve-lead ECG after VT termination indicated sinus rhythm **(C)**.

A 61-year-old female patient was admitted to our hospital with exertional dyspnea and lower-extremity edema for 3 months. The patient’s past medical history revealed hypertension for over 20 years, treated with oral nifedipine. Six years ago, the patient was admitted to a local hospital with several episodes of syncope. The patient noted feeling palpitation accompanied by perspiration and chest tightness; the symptoms had been recurrent. After admission to the local hospital, the patient underwent electrocardiogram (ECG) examination, echocardiography, and ventricle angiography. The ECG suggested VT and the ventricle angiography showed dilated right ventricle (LV) (Figure 1). Echocardiography revealed normal LV size and function. The initial diagnoses made by the local hospital were cardiomyopathy (undefined class) with VT. To reduce the risk of SCD, the patient received a single-chamber implantable cardioverter-defibrillator (ICD) at the local hospital. A single lead was also implanted in the RV (Supplementary Figure 1). Four years after the ICD implantation, the patient felt palpitations accompanied by discomfort in the precordial area. The ICD program recorded an attack of VT, which was terminated by ICD shock. The patient had no recurrence of such symptoms before admission to our hospital since then.

**FIGURE 2:**
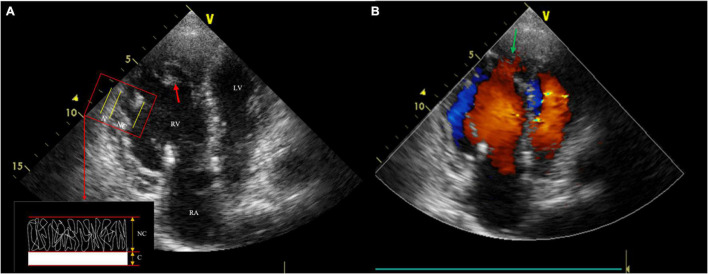
Transthoracic echocardiography (TTE) shows three anatomical components, including thin compacted layer, prominent intracardiac trabeculae (the red arrow) and deep intertrabecular recesses. The measured ratio of non-compacted to compacted myocardium was 2.7:1 **(A)**; Color Doppler echocardiography reveals deep recesses communicated with the Right ventricle (RV) cavity (the green arrow) **(B)**.

After admission to our hospital, physical examination showed severe pitting lower extremity edema. The laboratory test results were as follows: NT-proBNP, 1930.84 pg/ml (limit of reference, 0–125 pg/ml); troponin I, 472 ng/L (0–30.9 ng/L); fibrin degradation products, 49.33 μg/ml; and D-dimer, 17.23 ug/ml (0–0.5 μg/ml). Transthoracic echocardiography (TTE) revealed enlarged RV (RA, 36 mm; RV, 46 mm), right heart failure (tricuspid annular plane systolic excursion 9 mm, Tei index 0.27, RV-fractional area changes 22%), and typical features of spongy heart in the RV, including protruding trabecular muscles and deep intertrabecular recesses communicated with the RV cavity. Furthermore, the measured ratio of non-compacted to compacted myocardium (NC/C) was 2.7:1 (Figure 2). These findings suggested non-compaction in the RV. Furthermore, there were systolic blue regurgitation signals detected at the tricuspid orifice with a Vmax of 2.7 m/s, indicating tricuspid insufficiency (TI).

**FIGURE 3:**
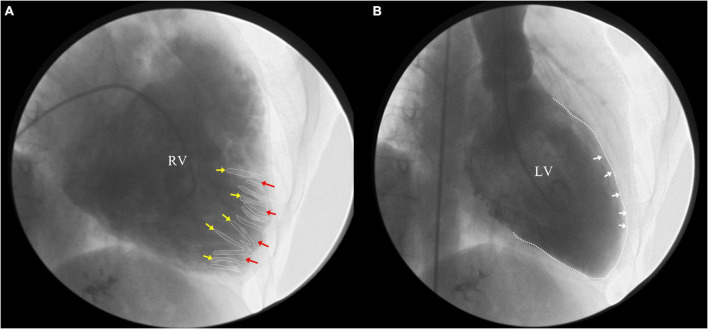
Right ventricle (RV) angiography revealed a dilated RV, excessive trabeculae and deep recesses with the retention of contrast agent at the apex and outflow tract of RV **(A)** (yellow arrow: trabeculae; red arrow: deep intertrabecular recesses), while no abnormality in left ventricle (LV) **(B)**.

Ensuing, we reviewed the videos of coronary angiography from the local hospital, which demonstrated a smooth intima and no stenosis, excluding the possibility of obstructive coronary artery disease (Supplementary Figure 2). Ventricular angiography revealed a dilated RV, excessive trabeculae, and deep recesses with the feather-like retention of the contrast agent at the apex and outflow tract of RV. No abnormality was observed in the LV (Figure 3). However, the characteristic features of RVNC failed to attract the attention of the local doctors.

**FIGURE 4:**
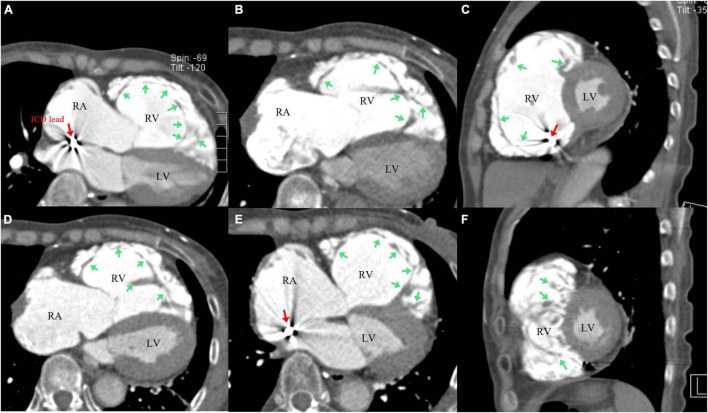
Contrast Computed tomography (CT) revealed abnormally dilated right ventricle (RV) and right atrium (RA), multiple protruding low-density intracardiac trabeculae attached to wall of the RV, and the high-density contrast agent in intertrabecular recesses and RV cavity **(A–F)** [green arrow: trabeculae **(A–F)**; red arrow: ICD lead **(A,C,E)**]; The left ventricle (LV) myocardium is thickened, and the trabeculae is normal **(A–F)**.

Due to the ICD, cardiac magnetic resonance (CMR) was not feasible; it was then substituted by Contrast Computed tomography (CT). The Contrast CT revealed multiple, protruding, low-density intracardiac trabeculae, high-density contrast agent in the intertrabecular recesses and RV cavity. The LV myocardium is slightly thickened, and the trabeculae is normal (Figure 4). Genetic testing identified a novel mutation (c.16799T > C/p.L5525S) in the exon 58 of titin gene (*TTN*, Ref Seq NM_001256850), which was predicted to be probably damaging by *in silico* prediction tools Polyphen2. The genetic implications further established the diagnosis of RVNC. Finally, isolated RVNC was diagnosed correctly by imaging and genetic testing results.

**FIGURE 5:**
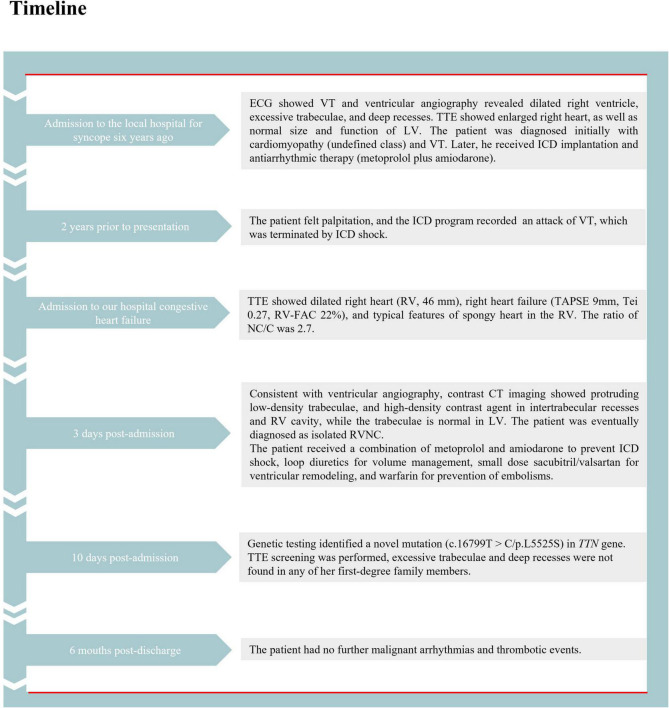
The detailed timeline of this patient from the symptom onset to follow-up. ECG, electrocardiogram; VT, ventricular tachycardia; TTE, transthoracic echocardiography; ICD, implantable cardioverter-defibrillator; RV, right ventricle; CT, computed tomography; LV, left ventricle; RVNC, right ventricular non-compaction.

Subsequently, the patient was treated with loop diuretics for volume management, oral sacubitril/valsartan sodium for ventricular remodeling, amiodarone and metoprolol for arrhythmia and ICD shock prevention, and warfarin for embolism prevention. The patient’s clinical symptoms markedly improved during hospitalization and within the 6 months of follow-up. The detailed timeline of this patient from symptom onset to follow-up is illustrated in Figure 5.

## Discussion

Isolated RVNC is not commonly reported. Ventricular non-compaction is associated with life-threatening complications, such as VT and systemic thromboembolism. The diagnosis of ventricular non-compaction was not considered by the local hospital when the patient manifested several episodes of syncope. Fortunately, the patient was treated in time and an ICD implantation was conducted to reduce the risk of SCD. The diagnostic criteria for ventricular non-compaction rely on non-invasive imaging examinations, such as TTE and CMR, which focus on the trabecular in the ventricle and the ratio of NC/C. At present, there are no diagnostic criteria for RVNC, and the diagnostic criteria for left ventricular non-compaction (LVNC) are mostly adopted clinically. Even so, overdiagnosis and overtreatment cannot be avoided. The prevalence rates in healthy populations who fulfill diagnostic criteria for LV non-compaction, proposed by Petersen et al. (3) (a ratio of NC/C greater than 2.3 at end-diastole) is up to 43%, raising concern about its disease status and the potential for overdiagnosis (4). Thus, population screening is generally ineffective. During the patient’s hospitalization, a final diagnosis of isolated RVNC was made by a combined examination of ventricular angiography, TTE, and CT. For the differential diagnosis, it is necessary to distinguish RVNC from arrhythmogenic right ventricular cardiomyopathy (ARVC). ARVC mainly involves the RV and is characterized by gradual replacement of myocardial tissue by fibrous fat. In our case, the imaging results indicated the structure of the RV was excessive trabeculae and deep recesses, which did not conform to the pathological changes of ARVC. CMR could visualize ventricular structure and the tissue characterization clearly enough to distinguish between the two diseases. However, CMR was not performed in our hospital because of the non-diamagnetic model of the patient’s ICD, and discontinuation of ICD during CMR scanning would put the patient at risk of ventricular tachycardia/fibrillation. A lack of CMR results is indeed a limitation of our case report. This case illustrates the crucial role of combined imaging examinations in the diagnosis of rare cardiomyopathy.

As the proband, the patient declared no history of syncope or SCD in her family. TTE screening was performed and no characteristic excessive trabeculae and deep intertrabecular recesses were found in any of the first-degree family members. Despite this, genetic testing in the setting of pathological non-compaction may contribute to the exact diagnosis and identification of at-risk relatives. Recently, accumulating studies have reported the genotype-phenotype correlations in patients with LVNC (2, 5). Ventricular non-compaction phenotype has been associated with more than 70 genes, such as *MYH7, ACTC1, MYBPC3, TNNT2, TPM1*, and *TTN* (6, 7). Among them, sarcomere genes, relevant for the structure of contractile and non-contractile elements with single missense mutations, are most commonly detected, accounting for the majority of genetic etiology of ventricle non-compaction (2, 8). The sarcomere gene consists of *MYH7, MYBPC3, TTN, ACTC1*, and so on (9). A meta-analysis indicated that the most frequently mutated genes in patients with LV non-compaction were *TTN* (11%), showing a pooled frequency of 11% (95% CI 4–29%) (10). In addition, sporadic cases of LVNC are prevalent in adults, indicating the contribution of acquired causes (2). Hence, the presumed mechanism of right heart failure in our patient is that the *TTN* gene missense mutation may terminate the compaction process of endocardial muscle trabecular muscle and result in non-compaction in RV, leading to reduced RV systolic function and severe right heart failure.

At present, the association between the clinical prognosis and genetics of non-compaction in adults remains controversial. Previous follow-up data on individuals with LVNC suggested no correlation between increased trabeculation and the development of diseases or adverse events (2, 11, 12). However, other evidence indicated carriers of pathogenic variants were more likely to develop adverse outcomes compared with non-carriers (58.5% vs. 25.8%; *P* < 0.01), and close follow-up for carriers was beneficial (1). Differences in screening genes and end points might partially explain the difference in these controversial results between these studies. Determining whether gene testing in adults with non-compaction can contribute to diagnostic issues and whether it benefits clinical treatment remains to be a major challenge for cardiologists.

Currently, there is no effective treatment for ventricular non-compaction. Strategies of therapy for confirmed patients are mainly targeted at the three major complications: heart failure, arrhythmia, and thromboembolism. After ICD implantation and pharmaceutical treatment as previously discussed, the symptoms of our patient improved significantly, with a good prognosis.

## Conclusion

We report a rare case of isolated RVNC that survived several episodes of syncope caused by VT. The patient was treated in time and correctly diagnosed through multi-imaging examinations and genetic testing. We recommended comprehensive cardiac screening for family members for earlier diagnosis and identification of at-risk relatives.

Isolated RVNC is rarely reported in current literature, causing possible underestimation of the incidence and severity of RVNC. Therefore, it is essential to identify and diagnose RVNC in clinical work.

## Data Availability Statement

The original contributions presented in the study are included in the article/Supplementary Material, further inquiries can be directed to the corresponding author/s.

## Ethics Statement

Written informed consent was obtained from the patient for the publication of any potentially identifiable images or data included in this article.

## Author Contributions

All authors listed have made a substantial, direct, and intellectual contribution to the work, and approved it for publication.

## Conflict of Interest

The authors declare that the research was conducted in the absence of any commercial or financial relationships that could be construed as a potential conflict of interest.

## Publisher’s Note

All claims expressed in this article are solely those of the authors and do not necessarily represent those of their affiliated organizations, or those of the publisher, the editors and the reviewers. Any product that may be evaluated in this article, or claim that may be made by its manufacturer, is not guaranteed or endorsed by the publisher.
