# Supplementary material for: A Rare Case of Isolated Right Ventricular Non-compaction With the Novel TTN Mutation
Source: Front Cardiovasc Med. 2022 Apr 29;9:845973. doi: 10.3389/fcvm.2022.845973 (PMC9098832; doi:10.3389/fcvm.2022.845973)
Supplement: Supplementary file 1 [file Data_Sheet_1.docx]

Supplementary Material

# Supplementary Figures and Tables

## Supplementary Figures

**Supplementary Figure 1.** X-ray showed the lead of single-chamber ICD was implanted in the RV.

**Supplementary Figure 2.** Coronary angiography showed a smooth intima and no stenosis in the right coronary artery, left anterior descending artery, and the left circumflex artery.


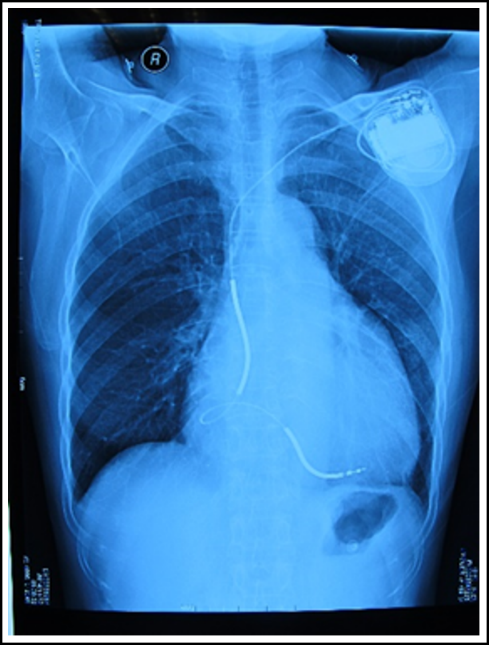


**Supplementary Figure 1.** X-ray showed the lead of single-chamber ICD was implanted in the RV.


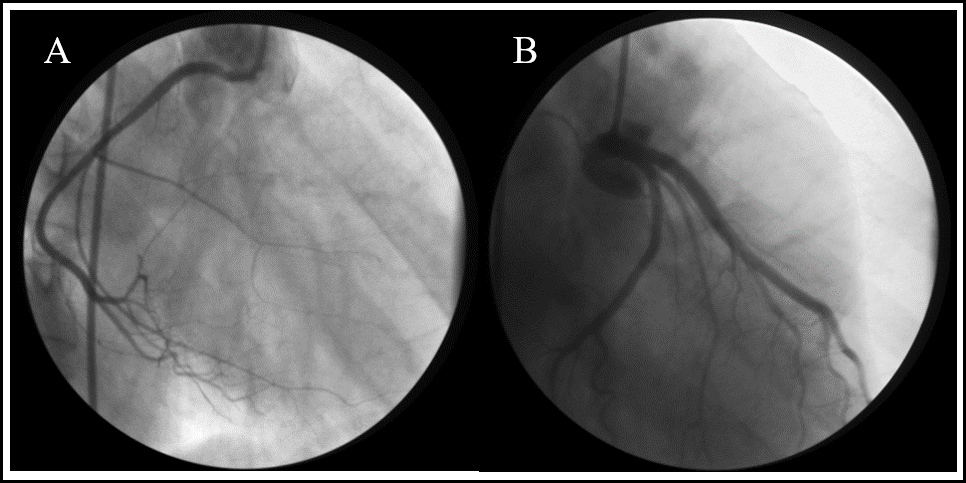


**Supplementary Figure 2.** Coronary angiography showed a smooth intima and no stenosis in the right coronary artery, the left anterior descending artery, and the left circumflex artery.
